# Experimental Investigation of the Flow and Heat Transfer Characteristics in Microchannel Heat Exchangers with Reentrant Cavities

**DOI:** 10.3390/mi11040403

**Published:** 2020-04-12

**Authors:** Binghuan Huang, Haiwang Li, Tiantong Xu

**Affiliations:** National Key Laboratory of Science and Technology on Aero Engines Aero-Thermodynamics, Beihang University, Beijing 100191, China; Huangbh@buaa.edu.cn (B.H.); 09620@buaa.edu.cn (H.L.)

**Keywords:** microchannel heat exchanger, reentrant cavity, heat transfer performance, pressure drop

## Abstract

The application of microchannel heat exchangers is of great significance in industrial fields due to their advantages of miniaturized scale, large surface-area-to-volume ratio, and high heat transfer rate. In this study, microchannel heat exchangers with and without fan-shaped reentrant cavities were designed and manufactured, and experiments were conducted to investigate the flow and heat-transfer characteristics. The impact rising from the radius of reentrant cavities, as well as the Reynolds number on the heat transfer and the pressure drop, is also analyzed. The results indicate that, compared with straight microchannels, microchannels with reentrant cavities could enhance the heat transfer and, more importantly, reduce the pressure drop at the same time. For the ranges of parameters studied, increasing the radius of reentrant cavities could augment the effect of pressure-drop reduction, while the corresponding variation of heat transfer is complicated. It is considered that adding reentrant cavities in microchannel heat exchangers is an ideal approach to improve performance.

## 1. Introduction

Heat exchangers are of great significance in chemical, energy, and electronics industries [1,2,3,4,5]. For recent decades, due to the continuously growing requirement for heat-transfer capacity, heat exchangers in conventional scale could not satisfy the demand. In contrast, microchannel heat exchangers have the advantages of larger surface-area-to-volume ratio and provide higher heat and mass transfer rate at smaller size and weight. Progressively, microchannel heat exchangers are being applied in cooling electronic systems, chemical machines, and aerospace equipment.

As proposed by Truckman et al. [6] in their pioneering work, a number of investigations have been conducted to explore the flow and heat-transfer characteristics of single-phase flow in microchannels. Previously, scholars paid attention to straight microchannels with smooth walls and investigated the influence of the cross-sectional shape, Reynolds number, and hydraulic diameter [7,8,9,10]. However, due to the rapid raising in the requirements for heat exchangers, modification in structure of microchannels is necessary to further improve the performance of heat transfer. Therefore, some researchers proposed to enhance the heat transfer via fabricating roughness elements within microchannels, which could disturb the flow. Herman et al. [11] studied the influence of curved vanes on the flow and heat transfer in microchannels. It was indicated that flow velocities in the groove region was accelerated and the heat transfer was enhanced. However, an obvious increase in the pressure drop was observed consequently. Dharaiva et al. [12] conducted a numerical analysis to investigate the flow and heat transfer in microchannels with 2D structured sinusoidal elements. Their work showed that, compared with smooth channels, the structured roughness elements on sidewalls enhanced heat transfer due to a combined effect of increased heat transfer area and modified flow, which resulted in an augment of pressure drop. Hong et al. [13] studied the flow and heat-transfer characteristics in microchannel heat sinks with offset strip-fin. When maximum wall temperature was set as constraint condition, it was demonstrated that strip-fin could enhance the heat transfer while the pressure drop was augmented simultaneously.

It is shown from these investigations that, compared with straight smooth microchannels, curved channels or structured roughness elements could disturb the flow and enhance the heat transfer. However, the disturbance results in a higher pressure drop in the microchannels. This would lead to higher pumping power consumption to drive the flow and deteriorate the economics of heat exchangers. Therefore, researchers attempt to explore alternative structures in microchannels to achieve heat-transfer enhancement at lower pressure drop. Reentrant cavity is a promising structure which attracts the interest of researchers. Xia et al. [14] analyzed the effect of triangular reentrant cavities on the flow and heat-transfer characteristics of microchannel heat sinks numerically. It was shown that, compared with straight microchannels, the heat transfer was enhanced for the whole range of Reynolds numbers they simulated, and the friction factor was reduced for the lower Reynolds numbers. Additionally, the influence of geometric parameters of triangular reentrant cavities was investigated, and the optimal ranges of these parameters were discussed. Ghani et al. [15] analyzed the characteristics of flow and heat transfer in a microchannel heat sink numerically. There were sinusoidal cavities and rectangular ribs on the sidewalls of microchannels. Their results showed that the combination of cavities and ribs could enhance the heat transfer and restrain the increase of the pressure drop. Pan et al. [16] conducted an experimental investigation to study the influence of geometric parameters of fan-shaped reentrant cavities. Their results indicated that microchannels with fan-shaped reentrant cavities were of better heat-transfer performance and lower pressure drop than straight ones. All these studies implied that reentrant cavities are of excellent performance in enhancing heat transfer, and its mechanism and performance in reducing pressure drop should be investigated thoroughly.

As aforementioned, it could be realized that internal structures are of significant impact on the heat transfer in microchannels. Moreover, in contrast to ribs or fins, which would result in a higher pressure drop than straight channels, reentrant cavities have shown the advantage by reducing the pressure drop. However, most of the relevant investigations were conducted numerically, and experimental study is limited. Besides, geometric parameters of reentrant cavities, which may affect the flow and heat transfer in microchannels, were not analyzed sufficiently. Motivated by these facts, in this work, test sections were manufactured, and experiments were conducted to analyze single-phase flow and heat-transfer characteristics in microchannels with fan-shaped reentrant cavities. The radius of reentrant cavities and Reynolds (*Re*) number were adopted as the main variables in experiments, to discuss their impact on heat-transfer enhancement and pressure-drop reduction.

## 2. Materials and Methods

### 2.1. Microchannels Fabrication

**Figure 1:**
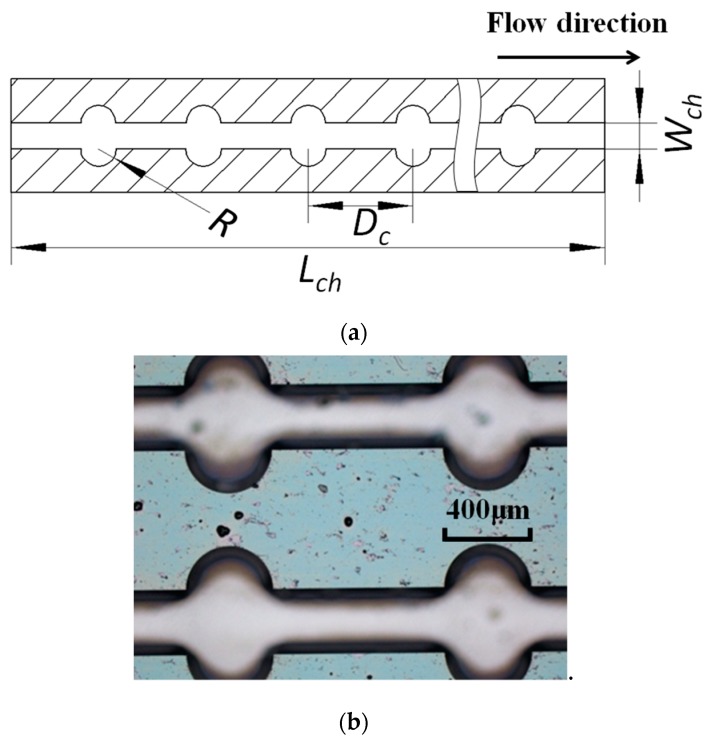
Structure of microchannels with reentrant cavities. (**a**) Structural parameters of a microchannel; and (**b**) Test section #4 observed with microscope.

The design of microchannels is illustrated in Figure 1. Ten parallel microchannels with an interval of 650 μm were etched on a silicon wafer (80 mm×15 mm×0.5 mm), as the thermal conductivity for silicon is 170 W/(m·K). Each microchannel was manufactured to be 300 μm in width ($W_{ch}$), 300 μm in height ($H_{ch}$), and 40 mm in length ($L_{ch}$); thus, the microchannel was 300 μm in hydraulic diameter ($D_{ch}$) with a square cross-section. Both ends of the microchannels were connected to plenums, which were 10 mm in length, 10 mm in width, and 300 μm in height. For test section #1, the microchannels were straight. For test sections #2, #3, and #4, 180° fan-shaped reentrant cavities were etched on sidewalls of all the microchannels. There were 128 pairs equidistance reentrant cavities with a radius (*R*) of 50 μm ($\alpha =R/D_{ch}=0.167$) in each channel of test section #2, and 64 pairs equidistance cavities with a radius of 100 μm in each channel of test section #3 (α=0.333), and then 32 pairs equidistance cavities with a radius of 200 μm in each channel of test section #4 (α=0.667), respectively. The ratio $\beta =R/D_{c}=0.167$ was kept the same for these three test sections, where $D_{c}$ represents the distance between two pairs of adjacent cavities.

The microchannels were manufactured with the dry etching-based micro-fabrication technologies, which were adopted in manufacturing single-crystal silicon for micro-electromechanical systems (MEMS). This process could achieve high vertical aspect ratios to obtain a square cross-section. Then the wafer was further processed to strip oxide layer. The etched side of the wafer was bonded with a silicon cover plate, and inlet/outlet manifolds were glued on this cover plate.

### 2.2. Experimental Setup

**Figure 2:**
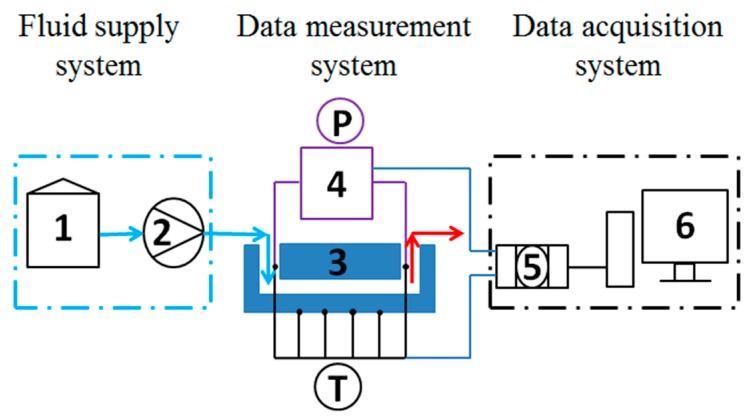
Schematic diagram of the experimental setup: (1) deionized water tank; (2) pump and flowmeter; (3) microchannel heat exchanger and film heater; (4) differential pressure transmitter; (5) data acquisition instruments; and (6) computer.

The schema of the experimental system is shown in Figure 2. The system consisted of a fluid supply circuit, a microchannel heat exchanger, a film heater, a differential pressure transmitter, and a data acquisition system. Deionized water was adopted as working fluid, and the flow rate was controlled by integrated system in the pump within an error of 0.5%. The Reynolds number (*Re*) varied from 40 to 120 in each microchannel. The inlet, outlet, and wall temperatures were measured by OMEGA T-type thermocouples, with a precision of 0.1 °C. The thermocouples were calibrated before experiments, and the resolution ranged from 0.0424 to 0.0428 mV/°C; hence, the average resolution adopted in calculation was 0.0426 mV/°C, within an error of 0.5%. The voltages of thermocouples were collected via a data acquisition system, which was composed of data acquisition instruments and a computer. The pressure drop between inlet and outlet manifolds was measured by Rosemount differential pressure transmitter. The microchannel heat exchanger was heated by a film heater, and the average heat flux could be obtained as $2.22\times 10^{5}\;W/m^{2}$, according to the heating power and the effective heat-transfer area. Insulation was provided to minimize the heat loss of the test section and film heater. For the consideration of comparison among test sections, the experimental conditions were kept the same. 

## 3. Data Analysis

### 3.1. Hydraulic Performance

The Reynolds (*Re*) number in each microchannel is defined as follows:

(1) $$Re=\frac{\rho_{f}V_{ch}D_{ch}}{\mu_{f}}$$

where $\rho_{f}$ is the density of the fluid, $V_{ch}$ is the average velocity in the microchannel, $D_{ch}$ is the hydraulic diameter of microchannel, and $\mu_{f}$ is the dynamic viscosity of the working fluid. The properties $\rho_{f}$ and $\mu_{f}$ were determined by the room temperature; and $V_{ch}$ was calculated according to the geometric parameters of microchannel and volumetric flow rate.

The hydraulic diameter of microchannel is defined as the ratio of fourfold cross-sectional area to the wetted perimeter. For a microchannel with rectangular cross-section, the hydraulic diameter could be obtained as follows:

(2) $$D_{ch}=\frac{4W_{ch}H_{ch}}{2\left(W_{ch}+H_{ch}\right)}$$

where $W_{ch}$ and $H_{ch}$ represent the width and height of the microchannel, which were measured with a microscope.

The pressure drop, ΔP, measured in experiments could be divided into pressure drop caused by friction inside the channel, and extra pressure loss due to passage configuration. 

For a single channel, the pressure loss, $\Delta P_{loss}$, could be calculated basing on Equation (3). The first term represents the pressure loss due to the passage bend of manifolds at the inlet/outlet, and the second term represents the pressure loss caused by sudden contraction and expansion at the inlet/outlet of channel.

(3) $$\Delta P_{loss}=\frac{1}{2}\rho_{f}\left(V_{im}^{2}+V_{om}^{2}\right)K_{90}+\frac{1}{2}\rho_{f}V_{ch}^{2}(K_{c}+K_{e})$$

where $\rho_{f}$ is the density of the fluid; $V_{im}$ and $V_{om}$ are the average velocity in the inlet/outlet manifolds; and $V_{ch}$ is the average velocity in microchannel. $K_{90}$ represents the loss coefficient for 90° bend, which is recommended to be 1.2 [17]. $K_{c}$ and $K_{e}$ represent the contraction and expansion coefficients due to area change, and they could be calculated according to the equations provided in [18].

For the multichannel configuration adopted in this investigation, the fluid flows through an inlet manifold and enters into a plenum; then the fluid is divided into several parallel channels and collected into another plenum, on the other end of the channels, and finally leaves the test section through an outlet manifold. Considering that the plenums on both ends of the channel are of constant cross-sectional area and the average velocities in manifolds and plenums are much lower compared with that in microchannel, pressure loss in these zones is relatively small and could be neglected. The pressure loss, $\Delta P_{loss}$, could be denoted as follows:

(4) $$\Delta P_{loss}=\Delta P_{im}+\Delta P_{om}+\Delta P_{c}+\Delta P_{e}$$

where $\Delta P_{im}$ and $\Delta P_{om}$ are the pressure loss at the inlet/outlet manifolds due to the cross-sectional area change, while $\Delta P_{c}$ and $\Delta P_{e}$ are pressure loss caused by the abrupt contraction and expansion at the inlet/outlet of microchannel, respectively.

$\Delta P_{im}$, $\Delta P_{om}$, $\Delta P_{c}$ and $\Delta P_{e}$ could be obtained according to the following equations [19,20]:

(5) $$\Delta P_{im}=\left(1-\sigma^{2}+K_{im}\right)\times \frac{1}{2}\rho_{f}V_{im}^{2}$$

(6) $$\Delta P_{om}=\left(\frac{1}{\sigma^{2}}-1+K_{om}\right)\times \frac{1}{2}\rho_{f}V_{om}^{2}$$

(7) $$\Delta P_{c}=\left[1-\sigma^{2}+0.5\left(1-\sigma \right)\right]\times \frac{1}{2}\rho_{f}V_{ch}^{2}$$

(8) $$\Delta P_{e}=\left[\frac{1}{\sigma^{2}}+\left(1-\sigma^{2}\right)\right]\times \frac{1}{2}\rho_{f}V_{ch}^{2}$$

where σ represents the small-to-large cross-sectional area ratio. $K_{im}$ and $K_{om}$ represent the pressure-loss coefficients at the inlet/outlet manifolds; their values are 0.22 and 0.64 for this design. Moreover, $\rho_{f}$ is the density of the fluid; $V_{im}$ and $V_{om}$ are the average velocity in the inlet/outlet manifolds; and $V_{ch}$ is the average velocity in channel.

For laminar flow in a microchannel with rectangular cross-section, the pressure drop due to friction could be calculated as follows [19]:

(9) $$\Delta P_{ch}=\frac{3\mu_{f}L_{ch}V_{ch}}{b^{2}F\left(a/b\right)}$$

where a refers to half of the width ($a=\frac{1}{2}W_{ch}$); b refers to half of the height ($b=\frac{1}{2}H_{ch}$) of the microchannel; and F(a/b) represents a function depended on the aspect ratio, which is approximately 0.42 for the square cross-section. It is indicated by this equation that the pressure drop is proportional to the dynamic viscosity of the fluid ($\mu_{f}$), the length of the microchannel ($L_{ch}$), and the average velocity inside microchannel ($V_{ch}$), and it is inversely proportional to $b^{2}$ and F(a/b). 

The length of microchannels tested is sufficient to neglect the additional loss in the developing flow region (Yun et al. [21] indicated that this additional loss could be neglected when the ratio of channel length to hydraulic diameter ($L_{ch}/D_{ch}$) is larger than 70). The apparent friction factor could be estimated by using the following equation [20]:

(10) $$f=\frac{2\Delta P_{ch}D_{ch}}{L_{ch}\rho_{f}V_{ch}^{2}}$$

Therefore, the pressure drop, $\Delta P_{total}$, measured by a differential pressure transmitter in this investigation, is mainly composed of the following parts:

(1) Pressure drop caused by friction inside the microchannel.
(2) Extra pressure loss at the inlet/outlet of microchannel on account of the abrupt contraction and expansion.
(3) Extra pressure loss on account of the passage bends of manifolds.
(4) Extra pressure loss at the inlet/outlet manifolds on account of the cross-sectional area change.

Additionally, extra pressure loss caused by friction in manifolds and plenums is relatively small and negligible, due to the much lower fluid velocity in these zones compared with that in microchannel. Part (1) is represented by$\;\Delta P_{ch}$, and the sum of Part (2)–(4) is represented by$\;\Delta P_{loss}$.

(11) $$\Delta P_{total}=\Delta P_{ch}+\Delta P_{loss}$$

### 3.2. Thermal Performance

The energy absorbed by the fluid could be estimated as follows:

(12) $$Q_{absorb}={\dot{m}}_{f}c_{f}\left(T_{f,out}-T_{f,in}\right)$$

where ${\dot{m}}_{f}$ represents the mass flux; $c_{f}$ represents the specific heat capacity of the fluid; and $T_{f,out}$ and $T_{f,in}$ denote the outlet and inlet temperature of the fluid.

The local convective heat-transfer coefficient of the microchannels could be obtained as follows:

(13) $$h=\frac{Q_{absorb}}{A_{tr}\left(T_{w}-T_{f}\right)}$$

where $T_{f}$ is the characteristic temperature of the fluid, which equals to the average of $T_{f,out}$ and $T_{f,in}$; and $T_{w}$ is the temperature of the wall and could be calculated by Fourier’s law, as follows:

(14) $$T_{w}=T_{ave}-\frac{qy}{\lambda_{Si}}$$

where $T_{ave}$ is the average of the temperature measured by the four thermal couples settled on the lower cover of the substrate; *q* is the heat flux; *y* is the distance from the lower cover of substrate to the bottom of channels; and $\lambda_{Si}$ is the thermal conductivity of the silicon substrate. 

$A_{tr}$ is the total effective convective heat-transfer area, which depends on the heating approach. In this investigation, the lower cover of substrate is heated; thus, heat transfer occurs mainly at the bottom and the sidewalls. For the convenience in calculation and comparison, $A_{tr}$ could be estimated as follows [22]:

(15) $$A_{tr}=L_{ch}\left(W_{ch}+2\eta_{fin}H_{ch}\right)N$$

where *N* is the number of microchannels, and $\eta_{fin}$ is fin efficiency, which could be obtained as follows:

(16) $$\eta_{fin}=\frac{\tan h\left(mH_{ch}\right)}{mH_{ch}}$$

In this equation, *m* is the fin parameter and could be estimated as follows:

(17) $$m=\sqrt{\frac{2h_{fin}}{\lambda_{Si}W_{fin}}}$$

where $\lambda_{Si}$ denotes the coefficient of thermal conductivity of the substrate, $W_{fin}$ denotes the width of the fin, and $h_{fin}$ represents the surface heat transfer coefficient of the fin.

The Nusselt (*Nu*) number is calculated by Equation (18):

(18) $$Nu=\frac{hD_{ch}}{\lambda_{f}}$$

where $\lambda_{f}$ is the thermal conductivity of the fluid, which depends on the characteristic temperature, $T_{f}$.

### 3.3. Experiment Uncertainty

Experimental uncertainty resulted from the measurement errors, and it could be estimated according to the method described by Moffat [23]. For a given function *R*, which depends on a series of independent variables $x_{1},\;x_{2},\;x_{3},\cdots ,\;x_{n}$, its uncertainty ΔR could be evaluated as follows:

(19) $$\Delta R=\left[{\left(\frac{\partial R}{\partial x_{1}}\Delta x_{1}\right)}^{2}+{\left(\frac{\partial R}{\partial x_{2}}\Delta x_{2}\right)}^{2}+{\left(\frac{\partial R}{\partial x_{3}}\Delta x_{3}\right)}^{2}+\cdots +{\left(\frac{\partial R}{\partial x_{n}}\Delta x_{n}\right)}^{2}\right]$$

where $\Delta x_{1},\;\Delta x_{2},\;\Delta x_{3},\cdots ,\;\Delta x_{n}$ present the uncertainties of these independent variables.

In this investigation, the main measurement errors include the errors of flow rate, temperature, pressure drop, and hydraulic diameter of microchannels. The maximum uncertainties of these parameters are listed in Table 1.

## 4. Results and Discussion

### 4.1. Heat-Transfer Characteristics

As mentioned in Section 2, for test sections #1 to #4, the parameter α varies from 0 to 0.667, and the radius of reentrant cavities ranges from 0 to 200 μm. For microchannels in test section #1 with α=0, they are the same with a conventional straight microchannel. The heat flux is $2.22\times 10^{5}\;W/m^{2}$, and the room temperature is approximately 22 °C. For each test section, the *Re* number varies from 40 to 120, and the corresponding outlet temperatures of the fluid and convective heat-transfer coefficients are compared to analyze their heat transfer performance. 

**Figure 3:**
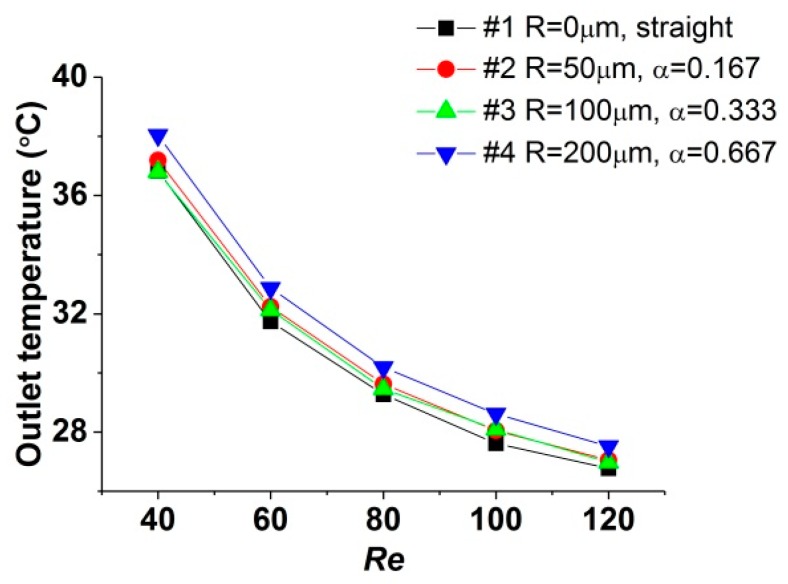
Influence of α on outlet temperature.

Figure 3 shows the comparison of outlet temperature versus the *Re* number. It is illustrated that the outlet temperature of test sections with reentrant cavities (#2, #3, and #4) is higher than that in straight one (test section #1), suggesting that reentrant cavities contribute to the enhancement of heat transfer. This effect could be explained from two aspects. Firstly, compared with conventional straight microchannels, the contact area between the sidewalls and the fluid is augmented due to the existence of cavities. Secondly, cavities could remodel the flow structure in microchannels, to enhance heat transfer. When the fluid enters a pair of cavities, the cross-section expansion causes spurting, and then as the fluid leaves the cavities, the cross-section contraction causes throttling. Both these two impacts would induce disturbances and secondary flow in microchannels, which could interrupt the hydraulic and thermal boundary layers. When the fluid enters the straight segments between two pairs of adjacent cavities, the hydraulic and thermal boundary layers would redevelop and then be interrupted in the next pair of cavities. This process of boundary layers’ destruction and redevelopment repeats periodically, resulting in enhancement of the heat-transfer performance. 

**Figure 4:**
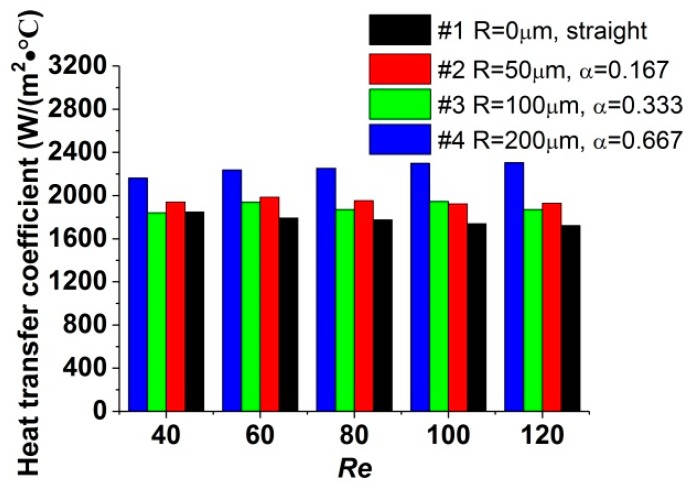
Influence of α on heat-transfer coefficient.

The convective heat-transfer coefficient for all the test sections is compared in Figure 4. For the range of the *Re* number investigated in this study, compared with straight microchannels, the heat transfer in microchannels with reentrant cavities is enhanced. As the *Re* number increases, the heat-transfer capacity for each test section rises by less than 7%, implying that the effect of *Re* number on the heat transfer is limited. In contrast, the radius of reentrant cavities has an obvious impact on the heat-transfer capacity. It is illustrated that, when α varies from 0.167 to 0.333, the convective heat-transfer coefficient decreases slightly. As α further increases from 0.333 to 0.667, the convective heat-transfer coefficient is augmented and is larger than that for α=0.167. This phenomenon suggests that a critical radius may exist, which would minimize the convective heat-transfer coefficient. It is considered that, when the fluid enters a pair of cavities, due to the expansion of cross-section, the mainstream would decelerate. Moreover, the fluid near the sidewalls would change flow direction and spurt into the cavities. This would cause separation at the expansive surface, which deteriorates the heat transfer, and impinge at the contractive surface, which enhances the heat transfer. When α varies from 0.167 to 0.333, both effects are augmented, and the deterioration is stronger than the enhancement; thus, the entire heat-transfer capacity decreases. As α increases from 0.333 to 0.667, the enhancement of heat transfer exceeds the deterioration, and an increase of the entire heat-transfer capacity is observed.

**Figure 5:**
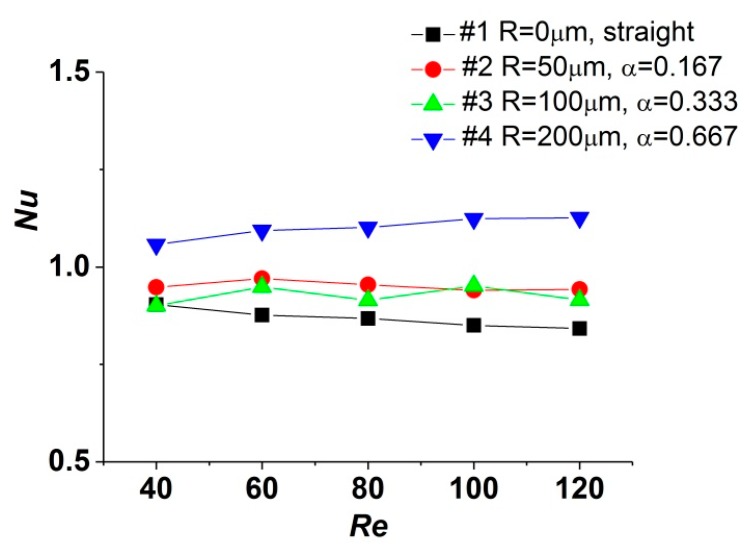
Influence of α on the *Nu* number.

The average *Nu* number calculated according to the experimental data is depicted in Figure 5. Compared with correlations proposed in [18] for developed laminar flow in conventional-scale straight channels, where the *Nu* number is constant (in channels with square cross-section, *Nu* = 3.61 for constant heat flux boundary condition and *Nu* = 2.98 for constant wall temperature boundary condition). When the *Re* number varies from 40 to 120, the curves of *Nu*–*Re* show different tendencies for these test sections.

In this study, the *Nu* number of test section #1 decreases by 6.83% as the *Re* number increases from 40 to 120. The similar phenomenon was also reported in [24,25], which was attributed to the axial and cross-sectional variation of fluid viscosity due to the steep temperature gradient. Meanwhile, the maximum variations of the *Nu* number in test sections #3 and #2 are 4.07% and 3.23%, implying that the decrease of the *Nu* number is restrained compared with that in test section #1. For test section #4, the *Nu* number increases by 6.53%. It is speculated that cavities with a larger radius could intensify the convection and mixture of fluid near the walls; thus, the temperature gradient is reduced. Therefore, the decrease of the *Nu* number could be restrained or even be converted into an increase. Moreover, the values of the average *Nu* number obtained in this study are much lower than those mentioned in [18], which was also observed in [26,27]. The *Nu* number could be considered as the ratio of conduction thermal resistance $D_{ch}/\lambda_{f}$ to convection thermal resistance 1/h. In contrast to channels in conventional scale, the hydraulic diameter of microchannels is much smaller; hence, the temperature gradient in the direction normal to the wall is steeper and the thermal conduction is more drastic. Therefore, the magnitudes of conduction thermal resistance are approximate to that of convection thermal resistance, indicating that the thermal conductivity in the fluid is important for heat transfer in microchannels.

According to the discussion above, it could be realized that microchannels with reentrant cavities show better heat-transfer performance than straight ones. The variation of the *Re* number could not affect the heat-transfer capacity obviously, while changing the radius of reentrant cavities could influence the heat transfer more significantly. 

### 4.2. Pressure-Drop Characteristics

Pressure drop is an important parameter in evaluating heat exchangers. Characteristics of pressure drop versus the *Re* number were investigated at room temperature (approximately 22 °C) when the test sections were unheated, a comparison was made with the data obtained when the test sections were heated at a heat flux of $2.22\times 10^{5}\;W/m^{2}$. The total pressure drop between inlet and outlet manifolds was measured by differential pressure transmitter directly, and the pressure drop in the microchannels was calculated according to these data and the equations mentioned in Section 3.1.

**Figure 6:**
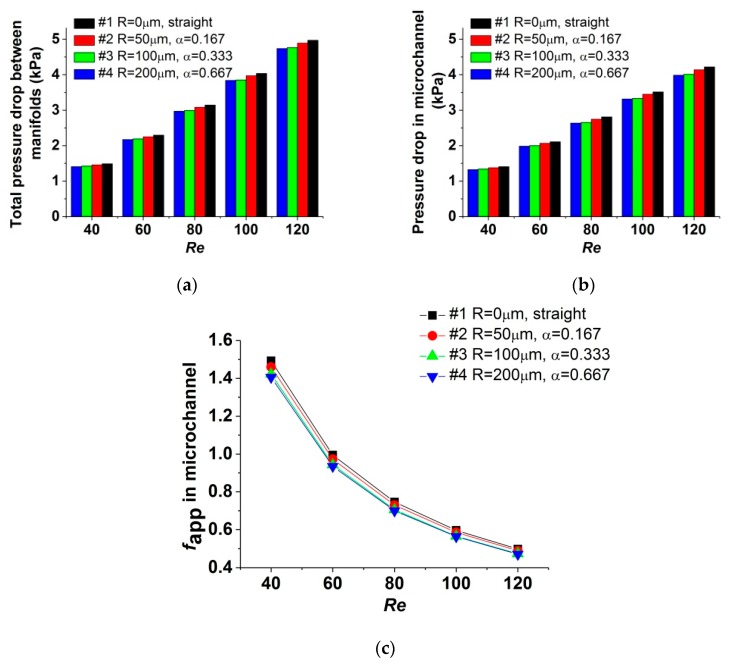
Influence of α on the pressure-drop characteristics when the test sections are unheated. (**a**) Total pressure drop between manifolds; (**b**) pressure drop in microchannel; (**c**) fapp in microchannel.

Figure 6a plots the total pressure drop of each test section when the test sections are unheated. Figure 6b plots the pressure drop in the microchannel, and Figure 6c shows the corresponding apparent friction coefficient (*f*_app_) for the data in Figure 6b. In Figure 6a, the pressure drop measured in test section #1 is in good agreement with theoretical value predicted by the equations in Section 3.1, within an error of 4.30%, which manifested that the precision of the experiment method is acceptable. It could be observed that, for the range of the *Re* number researched in this study, the pressure drop in test sections with reentrant cavities (test sections #2, #3, and #4) is reduced compared with that in the straight one (test section #1). For test sections #2, #3, and #4, as the radius of cavity increases, the effect of pressure-drop reduction is augmented. When the *Re* number varies from 40 to 120, compared with test section #1, the pressure drop in the microchannel is reduced by 1.85%~2.28% for test section #2, by 4.75%~5.49% for test section #3, and by 5.73%~6.22% for test section #4, demonstrating that the pressure-drop reduction is not influenced obviously by the variation of the *Re* number. The mechanism of pressure-drop reduction could be explained as that, for the flow in microchannels with reentrant cavities, the mainstream would slip over the liquid in cavities, and the friction resistance existed at this liquid–liquid interface is lower than that existed at the interface between mainstream and microchannel walls, which is a liquid–solid interface. For the test sections studied, increasing the radius of cavities could augment the effect of pressure-drop reduction. Besides, the expansion of the cross-section at the cavities decelerates the fluid, resulting in less of a pressure drop. For test sections #2 and #3, as the parameter *α* increases from 0.167 to 0.333, the effect of pressure drop reduction is enhanced obviously. However, for test sections #3 and #4, as the parameter *α* varies from 0.333 to 0.667, the distinction of pressure drop between them is less obvious. Considering that the ratio $\beta =R/D_{c}$ is kept the same for test sections #2, #3, and #4, the total length of the liquid–liquid interface is equal for these test sections. Moreover, the shape of the reentrant cavities for these test sections is also kept the same. This distinction could be attributed to the disturbance induced by the cross-section expansion at the cavities, which would cause increase in flow resistance. According to the present data, it is considered that further increase in the radius of reentrant cavities would result in limited augment in the pressure-drop reduction, due to the intensified disturbance. 

**Figure 7:**
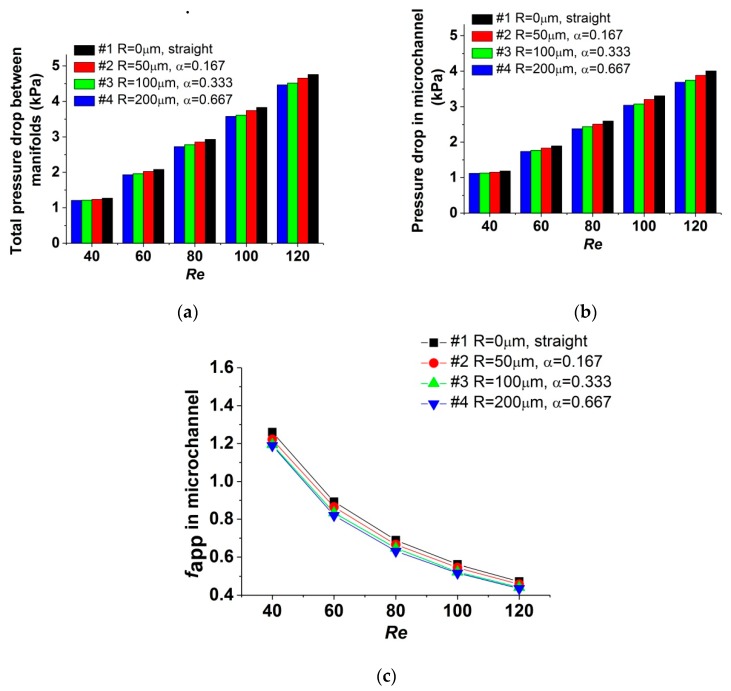
Influence of α on the pressure-drop characteristics when the test sections are heated. (**a**) Total pressure drop between manifolds; (**b**) pressure drop in microchannel; (**c**) fapp in microchannel.

Total pressure drop between the inlet and outlet manifolds, measured when the test sections are heated, is shown in Figure 7a, and the corresponding pressure drop in the microchannel is shown in Figure 7b; Figure 7c plots the apparent friction coefficient *f*_app_ for the data of Figure 7b. It could be observed that, compared with the data measured when the test sections are unheated, the effect of pressure-drop reduction is enhanced in the test sections with reentrant cavities. This could be attributed to the heat-transfer enhancement of reentrant cavities. The average temperature of the fluid in test sections #2, #3, and #4 is higher than that in test section #1. Because the fluid viscosity decreases with the increase of the fluid temperature, flow resistance and pressure drop are further reduced.

Heating could cause another influence on the pressure-drop characteristic, due to the distinction in fluid viscosity for different flow rate under the same heat flux. For a certain test section, when the *Re* number rises from 40 to 120, the flow rate required is tripled. However, the corresponding increase of heat-transfer capacity is not so obvious, and this is discussed in Section 4.1. Consequently, the average temperature of fluid would decrease with the *Re* number increasing, leading to a higher fluid viscosity compared with that for the lower *Re* number. Test section #4 could be taken as an instance. When the *Re* number varies from 40 to 120, the flow rate is augmented by 200%. When the test section is unheated, the pressure drop in the microchannel rises from 1.32 to 3.98 kPa, by 202%, which reflects a linear relationship between the pressure drop and the *Re* number. However, the pressure drop in the microchannel rises from 1.12 to 3.69 kPa, by 229%, when the test section is heated under a constant heat flux, and the increasing rate is amplified by 13.37%. This deviation caused by heating should be taken into consideration in the design and operation of microchannel heat exchangers.

Based on the analysis above, the conclusion could be drawn that, for a microchannel with reentrant cavities, the capacity of pressure-drop reduction is mainly influenced by the radius of reentrant cavities, while the distinction in fluid viscosity resulted from the variation of the *Re* number, which could not be neglected when the test sections are heated. 

### 4.3. Performance Analysis

For the consideration of evaluating the overall performance of the heat exchanger, the heat-transfer enhancement and corresponding pressure drop penalty should be taken into consideration simultaneously. Customarily, the thermal performance factor (*η*) is adopted as a criterion, which is defined as follows:

(20) $$\eta =\frac{Nu/Nu_{0}}{{\left(f_{\mathrm{app}}/f_{\mathrm{app},0}\right)}^{1/3}}$$

where $Nu_{0}$ and $f_{\mathrm{app},0}$ represent the *Nu* number and apparent friction factor of the straight microchannel (test section #1 in this study), respectively.

**Figure 8:**
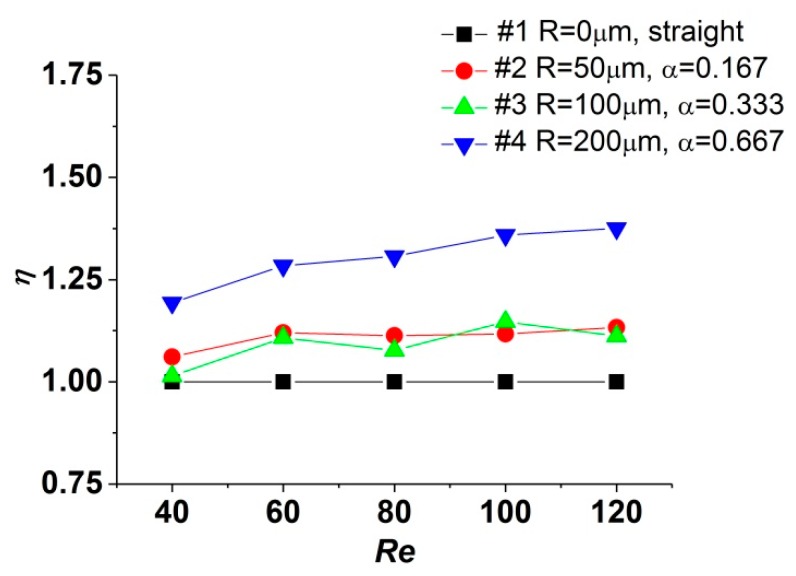
Influence of α on thermal performance factor (*η*).

Figure 8 depicts the variation tendency of *η* when the *Re* number increases. It could be realized that the values of *η* for test sections #2, #3, and #4 are higher than 1, indicating that the reentrant cavities could enhance the heat transfer in microchannels with lower augment of pressure drop, and the overall performance is improved compared with the straight one. When the *Re* number varies from 40 to 120, the values of *η* for test sections #2 and #3 increase by 6.78% and 9.62%, while that for test section #4 increases by 15.28%. As the *Re* number increases, due to the approximate tendency in the variation of friction factors, the characteristics of the *Nu* number determine the tendencies of the performance factor in different test sections. For the ranges of structural parameters and the *Re* number investigated, test section #4, with reentrant cavities in a radius of 200 μm, yields the best overall performance factor when *Re* = 120. 

## 5. Conclusions

In this study, microchannel heat exchangers with fan-shaped reentrant cavities were designed and manufactured. Experimental investigations were conducted to research the effect of cavity radius and the *Re* number on the flow and heat-transfer characteristics in microchannels. According to the experiment results, some conclusions could be drawn and summarized as follows:

(1) Compared with the straight one, the heat-transfer capacity in microchannels with reentrant cavities is enhanced. For the ranges of parameters analyzed in this study, the *Re* number has a limited impact on the heat transfer. When the radius of reentrant cavities increases, the variation of the convective heat-transfer coefficient is complicated and depends on the heat-transfer deterioration at the expansive surface and the enhancement at the contractive surface in cavities. For the range of radius investigated, it is considered that a critical radius may exist which could minimize the capacity of heat transfer.
(2) The *Nu* numbers of microchannels, with or without reentrant cavities, showed different tendencies as the *Re* number increased. Increasing the radius of reentrant cavities could enhance the mixture of the fluid near the walls and reduce the temperature gradient. Therefore, the decrease of the *Nu* number is restrained. Additionally, the *Nu* numbers obtained in this investigation are lower than theoretical values for conventional scale channels, indicating that the magnitudes of conduction thermal resistance and convection thermal resistance are approximate.
(3) The reentrant cavities could reduce the pressure drop in microchannels, compared with the straight one. The radius of cavities could significantly affect the capacity of pressure-drop reduction when the test sections are unheated. When the test sections are heated, the different heat-transfer capacity of test sections causes deviation in the fluid temperature and viscosity; thus, the effect of pressure drop reduction is further influenced.
(4) Compared with the straight one, the overall performance of microchannels with reentrant cavities is improved, due to the enhancement in heat transfer and the reduction in pressure drop. The radius of reentrant cavities and the *Re* number could affect the overall performance significantly. For the ranges of structural parameters and the *Re* number investigated, test section #4 yields the best overall performance factor when *Re* = 120.
(5) Due to the characteristics of enhancing heat transfer and reducing pressure drop, adding reentrant cavities in microchannel heat exchangers is an ideal approach to improve performance, especially for devices which are sensitive to the increase of pressure drop or pumping power consumption; hence, ribs or fins could not be adopted.

## Figures and Tables

**Table 1:** Parameters and estimated uncertainties.

Parameter	Maximum Uncertainty (%)	Parameter	Maximum Uncertainty (%)
Temperature (°C)	±0.5	Reynolds number	±1.74
Flow rate (mL/min)	±0.5	Friction factor	±3.58
Pressure drop (kPa)	±3.13	Heat transfer coefficient $W/(m^{2}\cdot$°C)	±1.33
Hydraulic diameter (μm)	±1.67	Nusselt number	±2.13

## Nomenclature

*a*	half of the width of the microchannel, μm
$A_{tr}$	total effective convective heat transfer area, $m^{2}$
*b*	half of the height of the microchannel, μm
$c_{f}$	fluid specific heat capacity, J/kg·°C
$D_{c}$	distance of two adjacent cavities, μm
$D_{ch}$	hydraulic diameter of microchannel, μm
*f_app_*	apparent friction coefficient
$f_{\mathrm{app},0}$	friction factor of the straight microchannel
F(a/b)	function depended on *a*/*b*
*h*	local convective heat transfer coefficient, $W/(m^{2}\cdot$°C)
$h_{fin}$	fin surface heat transfer coefficient, $W/(m^{2}\cdot$°C)
$H_{ch}$	height of microchannel, μm
$K_{90}$	pressure loss coefficient for a 90° bend
$K_{c}$	sudden contraction coefficient
$K_{e}$	sudden expansion coefficient
$K_{im}$	inlet manifold loss coefficient
$K_{om}$	outlet manifold loss coefficient
$L_{ch}$	length of microchannel, mm
*N*	number of parallel microchannels
*Nu*	Nusselt number
${Nu}_{0}$	*Nu* number of the straight microchannel
*m*	fin parameter
${\dot{m}}_{f}$	fluid mass flux, kg/s
*q*	heat flux, $W/m^{2}$
$\Delta P_{c}$	pressure drop caused by sudden contraction, Pa
$\Delta P_{ch}$	pressure drop in the microchannel, Pa
$\Delta P_{e}$	pressure drop caused by sudden expansion, Pa
$\Delta P_{im}$	pressure drop in the inlet manifold, Pa
$\Delta P_{loss}$	pressure loss, Pa
$\Delta P_{om}$	pressure drop in the outlet manifold, Pa
$\Delta P_{total}$	total pressure drop between inlet/outlet manifold, Pa
*R*	radius of reentrant cavities, μm
*Re*	Reynolds number
$T_{ave}$	average of the temperature measured by the four thermocouples, °C
$T_{f}$	fluid characteristic temperature, °C
$T_{f,in}$	fluid inlet temperature, °C
$T_{f,out}$	fluid outlet temperature, °C
$T_{w}$	Wall temperature, °C
*V*	total fluid volumetric flow rate
$V_{ch}$	average velocity in the microchannel, m/s
$V_{im}$	velocity in the inlet manifold, m/s
$V_{om}$	velocity in the outlet manifold, m/s
$W_{ch}$	width of microchannel, μm
*Greek letters*	
α	dimensionless parameter, (=*R*/$D_{ch}$)
β	dimensionless parameter, (=*R*/$D_{c}$)
$\rho_{f}$	fluid density, $\mathrm{kg}/m^{3}$
$\mu_{f}$	fluid dynamic viscosity, Pa·s
$\lambda_{f}$	fluid thermal conductivity coefficient, W/m·°C
$\lambda_{Si}$	thermal conductivity coefficient of silicon, W/m·°C
η	overall performance factor
$\eta_{fin}$	fin efficiency
σ	area ratio

## References

[1] Wang R., Wang J., Yuan E. (2019). Analysis and optimization of a microchannel heat sink with V-Ribs using nanofluids for micro solar cells. Micromachines.

[2] Liu Z., Qin S., Chen X., Chen D., Wang F. (2018). PDMS-PDMS micro channels filled with phase-change material for chip cooling. Micromachines.

[3] Mario Di Capua H., Escobar R., Diaz A.J., Guzmán A.M. (2018). Enhancement of the cooling capability of a high concentration photovoltaic system using microchannels with forward triangular ribs on sidewalls. Appl. Energy.

[4] Ling W., Zhou W., Yu W., Zhou F., Chen J., Hui K.S. (2019). Experimental investigation on thermal and hydraulic performance of microchannels with interlaced configuration. Energy Convers. Manag..

[5] Duan Z., Ma H., He B., Su L., Zhang X. (2019). Pressure drop of microchannel plate fin heat sinks. Micromachines.

[6] Tuckerman D.B., Pease R.F.W. (1981). High-performance heat sinking for VLSI. IEEE Electron Device Lett..

[7] Gunnasegaran P., Mohammed H., Shuaib N., Saidur R. (2010). The effect of geometrical parameters on heat transfer characteristics of microchannels heat sink with different shapes. Int. Commun. Heat Mass Transf..

[8] Weilin Q., Mala G.M., Dongqing L. (2000). Pressure-driven water flows in trapezoidal silicon microchannels. Int. J. Heat Mass Transf..

[9] Wang G., Hao L., Cheng P. (2009). An experimental and numerical study of forced convection in a microchannel with negligible axial heat conduction. Int. J. Heat Mass Transf..

[10] Tiselj I., Hetsroni G., Mavko B., Mosyak A., Pogrebnyak E., Segal Z. (2004). Effect of axial conduction on the heat transfer in micro-channels. Int. J. Heat Mass Transf..

[11] Herman C., Kang E. (2002). Heat transfer enhancement in a grooved channel with curved vanes. Int. J. Heat Mass Transf..

[12] Dharaiya V.V., Kandlikar S.G. (2013). A numerical study on the effects of 2d structured sinusoidal elements on fluid flow and heat transfer at microscale. Int. J. Heat Mass Transf..

[13] Hong F., Cheng P. (2009). Three dimensional numerical analyses and optimization of offset strip-fin microchannel heat sinks. Int. Commun. Heat Mass Transf..

[14] Xia G.D., Chai L., Wang H.Y., Zhou M., Cui Z. (2011). Optimum thermal design of microchannel heat sink with triangular reentrant cavities. Appl. Therm. Eng..

[15] Ghani I.A., Kamaruzaman N., Sidik N.A.C. (2017). Heat transfer augmentation in a microchannel heat sink with sinusoidal cavities and rectangular ribs. Int. J. Heat Mass Transf..

[16] Pan M.Q., Wang H.Q., Zhong Y.J., Hu M.L., Zhou X.Y., Dong G.P., Huang P.N. (2019). Experimental investigation of the heat transfer performance of microchannel heat exchangers with fan-shaped cavities. Int. J. Heat Mass Transf..

[17] Phillips R.J. (1987). Forced Convection, Liquid Cooled, Microchannel Heat Sinks. Master’s Thesis.

[18] Shah R.K., London A., Irvine T.F., Hartnett J.P. (1978). Laminar flow forced convection in ducts. Advances in Heat Transfer (Suppl. 1).

[19] Remsburg R. (2001). Thermal Design of Electronic Equipment.

[20] Shaughnessy E.J., Katz I.M., Schaffer J.P. (2005). Introduction to Fluid Mechanics.

[21] Yun H., Chen B., Chen B. Numerical simulation of geometrical effects on the liquid flow and heat transfer in smooth rectangular microchannels. Proceedings of the ASME International Conference on Micro/Nanoscale Heat and Mass (Transfer 3).

[22] Yin L.F., Jia L. (2016). Confined bubble growth and heat transfer characteristics during flow boiling in microchannel. Int. J. Heat Mass Transf..

[23] Moffat R.J. (1988). Describing the uncertainties in experimental results. Exp. Therm. Fluid Sci..

[24] Tso C.P., Mahulikar S.P. (1998). The use of the Brinkman number for single phase forced convective heat transfer in microchannels. Int. J. Heat Mass Transf..

[25] Tso C.P., Mahulikar S.P. (2000). Experimental verification of the role of Brinkman number in microchannels using local parameters. Int. J. Heat Mass Transf..

[26] Choi S.B., Barren R.R., Warrington R.Q. (1991). Fluid flow and heat transfer in micro-tubes. ASME DSC.

[27] Hetsroni G., Gurevich M., Mosyak A., Rozenblit R. (2004). Drag reduction and heat transfer of surfactants flowing in a capillary tube. Int. J. Heat Mass Transf..

